# Color Vision Losses in Autism Spectrum Disorders

**DOI:** 10.3389/fpsyg.2017.01127

**Published:** 2017-06-30

**Authors:** Elaine C. Zachi, Thiago L. Costa, Mirella T. S. Barboni, Marcelo F. Costa, Daniela M. O. Bonci, Dora F. Ventura

**Affiliations:** ^1^Department of Experimental Psychology, Nucleus for Neuroscience and Behavior, Institute of Psychology, University of São PauloSão Paulo, Brazil; ^2^Cognitive Neuroscience Laboratory and Developmental Disorders Program, Center for Health and Biological Sciences, Mackenzie Presbyterian UniversitySão Paulo, Brazil

**Keywords:** autism, high-functioning Autism, Asperger Syndrome, color vision, Cambridge Color Test, intelligence

## Abstract

Autism spectrum disorders (ASDs) are neurodevelopmental conditions characterized by impairments in social/communication abilities and restricted behaviors. The present study aims to examine color vision discrimination in ASD children and adolescents without intellectual disability. The participants were also subdivided in order to compare color vision thresholds of autistic participants and those who achieved diagnostic criteria for Asperger Syndrome (AS). Nine subjects with autism, 11 participants with AS and 36 typically developing children and adolescents participated in the study. Color vision was assessed by the Cambridge Color Test (CCT). The Trivector protocol was administered to determine color discrimination thresholds along the protan, deutan, and tritan color confusion lines. Data from ASD participants were compared to tolerance limits for 90% of the population with 90% probability obtained from controls thresholds. Of the 20 ASD individuals examined, 6 (30%) showed color vision losses. Elevated color discrimination thresholds were found in 3/9 participants with autism and in 3/11 AS participants. Diffuse and tritan deficits were found. Mechanisms for chromatic losses may be either at the retinal level and/or reflect reduced cortical integration.

## Introduction

Autism Spectrum Disorder (ASD) is a neurodevelopment disorder characterized by impairments in social interaction and social communication besides restrictive and/or repetitive patterns of behavior, interests or activities (American Psychiatric Association, 2013). The intensity of ASD symptoms is highly variable ranging from very severe to mild forms. It also includes a variability of intellectual functioning profiles ranging from high intelligence to intellectual disability (ID) (Klin, 2006; Crespi, 2016).

Under the autism spectrum, mild forms without intellectual disability have been known as high-functioning autism (HFA) (Klin and Volkmar, 1995), although the Diagnostic and Statistical Manual of Mental Disorders (DSM) and the manual of the International Classification of Diseases (ICD) do not provide its formal description (Montgomery et al., 2016). Asperger Syndrome (AS) was incorporated in the former 4th edition of the DSM (DSM-4; American Psychiatric Association, 1994) and in the current edition of ICD (ICD-10; World Health Organization, 1994) as a diagnostic category, which is distinguished from autism without ID by the absence of significant delays in receptive language or speech, cognitive development, self-help capabilities, and exploration of the environment in early stages of development (Klin, 2006). Individuals with AS exhibit average or superior intellectual quotient IQ, huge vocabulary and good grammar ability (Wing et al., 2011).

The differentiation of AS from autism without ID has been controversial and provoking disagreement among researchers, although the actual DSM-5 (American Psychiatric Association, 2013) removed the Asperger Syndrome as a diagnosis category, and included it into the larger classification entitled Autism Spectrum Disorders (ASDs), the view that profile differences between AS and autism without ID are not solely quantitative but also comprises qualitative issues have been supported (e.g., Tsai and Ghaziuddin, 2014) and suggests the need for further research.

In addition to the primary importance of social impairment for ASD characterization, sensory perceptual alterations are an aspect of interest in this condition. There is an extensive literature on the prevalence of sensory symptoms in ASD. The first descriptions refer to the early works by Kanner (1943), and by Hans Asperger 1 year later (Frith, 1992; Klin, 2006). The observation of abnormal responses to sensory stimulation in these individuals was reflected in the changes in DSM-5, which added sensory issues to the diagnostic criteria for ASD, recognizing that hypersensitivity or hyposensitivity to stimuli are frequent (Buxbaum and Baron-Cohen, 2013).

Vision alterations are a common feature described in autism and range from optometric issues, visual acuity and low-level visual processing (static and dynamic sine-wave contrast sensitivity, color, depth, stereopsis, motion), to aspects such as perceptual organization, reading, gaze detection, recognition of biological motion and of emotion in faces (reviewed by Simmons et al., 2009; Bakroon and Lakshminarayanan, 2016). Previous researches in ASD encompassed color processing in relation to other abilities or tasks such as reading, learning, speed and/or accuracy in searching for targets, and behavior toward color perception (obsession with a color and phobia of others; Ludlow et al., 2006, 2012, 2014; Wilkinson and McIlvane, 2013).

In a study that integrated color memory and color discrimination measured by the Ishihara Test and the City University Color Vision Test 3rd ed., autistic children with intellectual disability as well as children with moderate learning difficulties exhibited poor color discrimination in comparison to typically developing children (Heaton et al., 2008). In another work, autistic subjects without ID were less accurate in discriminating colored targets presented on chromatic backgrounds (Franklin et al., 2008). The same study showed that autism was also associated with diffuse color vision deficits in other experiment that used the Farnsworth–Munsell 100-hue test (FM100) plus a luminance control to assess task difficulty (Franklin et al., 2010). The errors of the group with ASD were higher than the control's and spread over the entire spectrum without a concentration on either the red-green or the blue-yellow axes. However, the luminance task appeared to be easier than the chromatic task for both groups and therefore was not considered an adequate control of task difficulty. To achieve better control of task difficulty, another experiment assessed luminance and chromatic discrimination performance using an adaptive psychophysical staircase procedure which varied stimulus conditions to achieve a fixed performance (82% correct responses) thus equating difficulty for both tasks. In this situation, the autistic group still had higher thresholds in color discrimination, but did not differ from controls in the luminance task (Franklin et al., 2010). Another study found children and adolescents with ASD (7–15 years) had significantly higher total error scores in the FM100 when compared to typically developing children aged 8–9 years (Cranwell et al., 2015).

Studies regarding color discrimination at the sensory level (early visual processing) on autism are scarce. Considering the high occurrence of sensory dysfunction in ASDs, the present work aims to evaluate color discrimination in these individuals, as well as to compare this function in autistic children/adolescent without ID and those who fulfill the DSM-4 criteria for AS. Color vision was assessed here using a rigorous computerized color vision test that has been applied very successfully in a number of clinical studies due to the fact that it can determine color discrimination thresholds accurately, in a very short time, with good reproducibility, both in children and in adults with different pathologies (Ventura et al., 2003; Castelo-Branco et al., 2004; Costa et al., 2006, 2007, 2012; Feitosa-Santana et al., 2008, 2010; Goulart et al., 2008; Moura et al., 2008; Lacerda et al., 2012; Gualtieri et al., 2013).

## Materials and methods

### Participants

**Figure 1:**
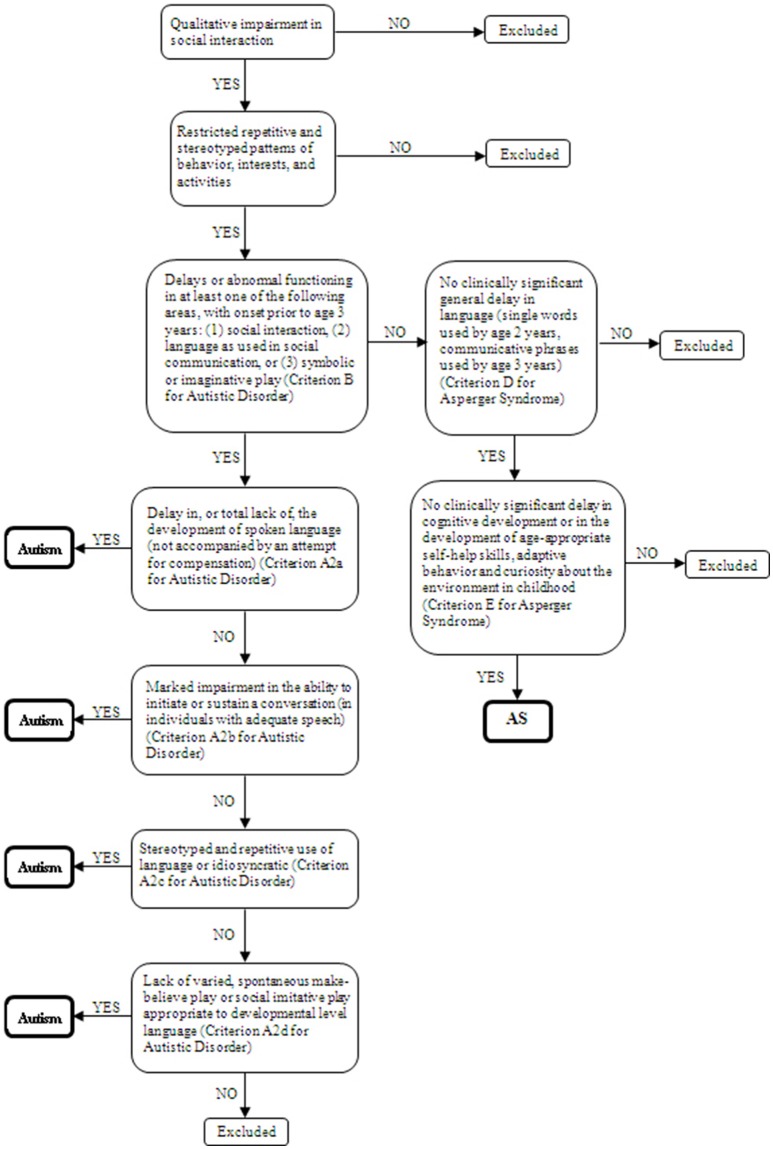
Decision tree based on the DSM-4 criteria for autism and Asperger Syndrome classification.

Twenty children and adolescents (6–19 years old) with diagnosis of ASDs as defined by the DSM-4 and 36 controls participated in the study. They had a clinician-assigned diagnosis of autism or AS with basis on the DSM-4-TR (American Psychiatric Association, 2000). Inclusion criteria of participants in the study were indication of autism disorder according to the Childhood Autism Rating Scale (CARS) (Schopler et al., 1986) (cutoff = 30) for the ASD participants and an estimated IQ equal or above 80 for all groups. Estimated IQ was based on Vocabulary and Block Design subtests of the Wechsler scales–WISC-III (Wechsler, 1991) for children and adolescents under 17 years and the WAIS-III (Wechsler, 1997) for the remaining participants. A structured anamnesis interview based on DSM-4-TR criteria (American Psychiatric Association, 2000) and carried out with the mother or both parents was used to assess presence of autism or AS. Group differentiation was made by one researcher at the time of the study with basis on the interview. As both conditions share the same characteristics namely: qualitative impairment in reciprocal social interaction (criterion A1 for Autistic Disorder and A for Asperger Syndrome) and restricted and repetitive stereotyped patterns of behavior interests, and activities (criterion A3 for Autistic Disorder and B for Asperger Syndrome) (Woodbury-Smith et al., 2005), DSM-4 criteria concerning delays or abnormal functioning in other areas were used for differentiating the groups. Figure 1 shows the decision tree used for the composition of the ASD subgroups. If parents had concerns prior to age 3 in terms of social interaction, social communication or symbolic/imaginative play (criterion B for Autistic Disorder), the potential participant was investigated for other impairments in communication. Any qualitative deficit in communication as defined by items “a” to “d” of the criterion A2 for Autistic Disorder lead the child/adolescent to be allocated in the group with autism without ID. On the other hand, participants that composed the group with AS did not support criterion B for Autistic Disorder, had no history of language development delays (single words used by age 2 years and communicative phrases used by age 3 years), and no significant delays in cognitive development, self-help skills, adaptive behavior, and curiosity about the environment. Only individuals whose the researcher's diagnosis was in agreement with the clinician were included in the study.

Initially, 10 participants had been allocated in the autistic group. However, 1 was excluded because was known to present congenital color vision deficiency. This child had very high thresholds in protan and deutan color confusion axis on Cambridge Color Test, in both eyes tested monocularly suggesting a congenital color vision deficiency. The highest threshold was in deutan axis. To evaluate if the reduction of color discrimination was due to congenital origin the subject was tested with the Ishihara pseudoisochromatic plates. The Ishihara test was performed with the 1981 edition of 24 pseudoishochromatic plates (Kanehara & CO., Tokyo, Japan). The plates were administered under fluorescent lamps (compact type) illumination that is known to provide a closer approximation to natural daylight. The subject was instructed to identify a number amidst luminance and spatial noise, using the hue as the only clue. The plates were presented at 1 m of distance, with average response time of 2–4 s. The subject failed to discriminate most of the plates and do not read correctly the plates except for the first control plate. The result confirms a congenital color vision deficiency on red-green axis. Indeed, the maternal grandfather of the subject had a diagnosis of congenital color vision deficiency, which explains the heritability of the color vision defect presented by the child.

Nine participants formed the group with autism (6–19 years old, 8 male) and 11 individuals were allocated to the AS group (6–13 years old, 11 male). The control group was formed by 36 typically developing children and adolescents (6–18 years old, 34 male). Controls had no evidence of developmental conditions as verified in the anamnesis. Background characteristics of the participants are presented in Table 1. Kruskal-Wallis test revealed no age significant differences among groups KW-H(2.56) = 0.98, *p* = 0.61. ASD participants and controls under 17 years old were tested on the Vocabulary and the Block Design subtests of the WISC-III. The remaining participants (2 participants with autism and 1 control) were submitted to the equivalent subtests of the WAIS-III. Individuals with AS showed higher estimated IQs [KW-H(2.44) = 5.80, *p* = 0.05] and Vocabulary scaled scores [KW-H(2.44) = 5.46, *p* = 0.06], but the differences between groups were marginal. Block Design scores did not differ among the 3 groups, KW-H(2.44) = 0.95, *p* = 0.62. Statistical analysis revealed no significant difference in CARS scores obtained by autistic or AS participants [KW-H(1.20) = 1.70, *p* = 0.19].

**Table 1:** Background characteristics of the groups.

	**Autistic group**	**AS group**	**Control group**	*p*-value^*^
N	9	11	36	–
Gender (M:F)	8:1	11:0	34:2	–
Age	8 (6–19)	9 (6–13)	9 (6–18)	0.61
IQ	100 (80–123)	112 (103–144)	106 (95–138)	0.05
Vocabulary	12 (4–17)	12 (9–19)	11 (8–14)	0.06
Block Design	10 (6–15)	13 (9–19)	10 (7–19)	0.62
CARS score	33.5 (31–34.5)	32.5 (30.5–34)	–	0.19

*N, number of valid cases; AS, Asperger Syndrome; IQ, intelligence quotient; CARS, Childhood Autism Rating Scale. Data concerning age, IQ, Vocabulary, Block Design and CARS scores are median (range)*.

* *p-value obtained by Kruskal Wallis test*.

Exclusion criteria for all groups consisted in history of traumatic brain injury or seizure. Children and adolescents with a known associated syndrome (e.g., tuberous sclerosis) were also excluded. This study was approved by the Ethics Committee of the Institute of Psychology of the University of São Paulo (authorization number 2009.045). This is part of a large study in which neuropsychological functions were evaluated. Parents of participants under 18 years old signed the informed consent. The adolescent with autism and the control who are aged 18 years gave their own consent. One autistic participant aged 19 years who was not judicially emancipated had parental consent.

### Color vision test and procedures

All participants had 20/20 or better best-corrected visual acuity assessed with an ETDRS chart (tumbling E) 3 m away from the subject.

Color vision discrimination was measured with the Cambridge Color Test (CCT) v2.0 (Cambridge Research Systems, Rochester, UK). The Cambridge Color Test (CCT) is a computerized tool that was proven to be effective in the detection and quantification of acquired Color vision losses (e.g., Ventura et al., 2003; Castelo-Branco et al., 2004; Costa et al., 2006, 2007, 2012; Feitosa-Santana et al., 2008, 2010; Moura et al., 2008; Lacerda et al., 2012; Gualtieri et al., 2013). The stimulus was generated in a high-resolution color monitor, Sony FD Trinitron model GDM-F500T9, Sony Corporation, Tokyo, Japan). The computer had a graphic board VSG 2/5 (Cambridge Research Systems, UK) attached to it. Monitor calibrations for luminance were performed using the OptiCAL 200-E photometer (Cambridge Research Systems) and the standard calibration procedures designated by the VSG Desktop library (version 8.0). The testing sessions were conducted in a dark room with blackout. The experimenter's monitor screen remained off and illumination was provided only by the monitor used to present visual stimuli. The participant was positioned at a distance of 3 m from the monitor, which was left on for at least 45 min before testing. The target stimulus generated by the CCT consisted of a Landolt “C” that differed in chromaticity from the background (coordinates u′ = 0.1977, v′ = 0.4689 International Commission on Illumination [CIE] 1976 color space). Stimulus and background luminance were measured using a Minolta CS1000 photometer. In one of the first papers on the CCT, Regan et al. (1994) reported the Landolt C gap subtended 1 degree of visual angle from a distance of 2.4 m between the subjects and the monitor. In the present study, the C gap size corresponded to 1.25° as a proportional value since the participants positioned 3 m away from the monitor, with the outer diameter 5.4° and the inner diameter 2.75°. Both stimulus and background were composed of small circles of 0.5–2 cm in diameter and six luminance levels (8, 10, 12, 14, 16, and 18 cd × m^−2^) randomly distributed in the screen in order to avoid possible luminance cues. For more information on the development of this test and the rationale behind pseudoisochromatic stimuli we refer to Reffin et al. (1991) and Regan et al. (1994).

The “C” stimulus was presented with its opening directed to one of the 4 sides of the screen (right, left, top, and bottom). The CCT employs a 4 alternative forced-choice strategy that requires the subject to press one of the 4 buttons of a response box to indicate the position of the gap in the “C.” It was used the button box provided by Cambridge Research Systems. The participants were asked to keep their hands and the button box on their legs in a position that was favorable for detection of the signal by the receiver. Color discrimination thresholds were estimated by a psychophysical staircase procedure with a dynamic step size. The staircase began with a hue with chromaticity coordinates for the target stimulus with the maximum achievable color within the monitor's gamut, which is within the range of the CIE u′v′1976 chromaticity diagram. The distance between target and background decreased along a line connecting the two, every time the subject made a correct response and increased when the response was incorrect (1 up/1 down staircase rule with a step size of 20% of the average chromaticity). The step size was changed dynamically by proprietary software from the test manufacturer. After 12 response reversals the CCT software determined the threshold for each vector as the average chromaticity of the six final staircase reversals (for additional details see Regan et al., 1994). The results are expressed in u′, v′ coordinates in CIE 1976 Color space.

The Trivector protocol of the CCT was used. It determines color vision thresholds along the protan, deutan and tritan confusion lines. The 3 corresponding staircases are administered interchangeably and randomly in the same session. The examiner was blind to the grouping condition of participants (autism without ID or AS).

In order to consider effects of slowness on elevated CCT discrimination thresholds, the response latencies of ASD participants with high color vision thresholds were measured using the Rapid Visual Information Processing (RVP). The RVP is part of the Cambridge Neuropsychological Test Automated Battery (CANTAB) (Cambridge Cognition, 2005) and requires detection of a 3 digit target sequence presented at computer screen in which digits appear at the rate of 100 digits/min. The responses are registered using a button box. The CANTAB software compares the subjects' results with normative data. The RVP Mean Latency measure was adopted as a parameter of response speed.

### Data analysis

Color discrimination thresholds values obtained by the control group showed adhesion to the normal distribution with the Kolmogorov-Smirnov test. Color vision defect among ASD individuals were determined using tolerance limits based on controls' color vision thresholds. Statistical tolerance limits are boundaries of a statistical tolerance interval estimated based on a sample covering a specified proportion for a determined function under study in the population. In the present study, the upper limits of two-sided tolerance intervals for 90% of the population and a probability of 90% were calculated (Howe, 1969). Upper tolerance limits were established with the equation: UTL = mean + K^*^SD, where the mean and SD of the control group thresholds were used and the value of K for 90% of the population with a probability of 90% was 1.866. A descriptive analysis was also performed to compare the participants' thresholds with standard limits for young adults provided by CCT handbook that are 100 (protan), 100 (deutan) and 150 (tritan).

The Yates' Corrected Chi-Square was used for comparison of frequency of color vision losses between participants with autism and AS. Color vision thresholds were compared among the groups using the non-parametric Kruskal-Wallis test with subsequent Mann-Whitney *U*-tests as *post hoc* analyses. For this purpose, each of the 3 groups was subdivided into broader age clusters (6–10 years old and 11–19 years old). Spearman's correlation tests were used to examine associations between color vision results and IQs and ages. Analyses were performed with the software Statistica (StatSoft v.11.0). A *p*-value below 0.05 was considered statistically significant.

## Results

Table 2 presents mean's color discrimination thresholds in the CCT, expressed in vector length and in units u′v′ 1976 CIE diagram, obtained by the 36 control participants along the protan, deutan, and tritan color confusion axes. The values were rounded off to the nearest integer number. Control participants showed nearby values for protan (59, *SD* = 16) and deutan (59, *SD* = 17) axes, and the highest mean for the tritan axis (88, *SD* = 31). The protan, deutan, and tritan axes had an upper tolerance limit of 91, 92, and 149, respectively.

**Table 2:** Color discrimination thresholds obtained by the control group (*n* = 41) and respective upper tolerance limits calculated for 90% of the population with 90% probability.

	**Protan axis**	**Deutan axis**	**Tritan axis**
Thresholds^*^ (mean ± SD)	59 ± 16	59 ± 17	88 ± 31
Upper tolerance limit	91	92	149

*SD, standard deviation*.

* u′v′^*^10^3^ vector length units of the 1976 CIE chromaticity diagram.

Individual color discrimination thresholds are shown in Table 3. The upper tolerance limits, based on the thresholds obtained with the control group were used to assess color vision performance. Color discrimination thresholds that exceeded the value of the upper tolerance limit evidenced impairment in the color discrimination capacity. Losses could be found at all three color confusion axes, in which case the defect is considered a diffuse color vision defect. Conversely, the losses could be selectively located at either the red-green dimension, i.e., in the protan and deutan axes, or at the blue dimension, in the tritan axis.

**Table 3:** Color discrimination thresholds of participants with autism or AS, and controls in u′v′^*^10^3^ units.

	**Age**	**Protan axis**	**Deutan axis**	**Tritan axis**
**AUTISTIC GROUP (*N* = 9) SUBJECT**				
1	6	44	49	86
2	7	47	73	58
3	7	72	53	323^*^
4	7	88	44	92
5	8	105^*^	130^*^	249^*^
6	14	51	65	167^*^
7	15	39	48	63
8	18	47	73	139
9	19	35	33	44
**AS GROUP (*****N*** = **11) SUBJECT**				
10	6	89	71	107
11	6	119^*^	125^*^	206^*^
12	6	84	119^*^	273^*^
13	6	129^*^	457^*^	255^*^
14	8	70	42	89
15	9	70	42	89
16	9	87	66	46
17	11	55	29	71
18	11	42	36	61
19	12	63	50	93
20	13	32	32	35
**CONTROL GROUP (*****N*** = **36) SUBJECT**				
21	6	58	67	113
22	6	70	66	136
23	6	80	90	151^*^
24	6	88	48	134
25	7	33	41	65
26	7	70	40	107
27	7	71	71	101
28	7	60	62	108
29	7	62	36	101
30	8	73	81	71
31	8	56	42	84
32	8	66	44	77
33	8	47	62	94
34	8	49	61	88
35	8	47	39	23
36	8	93^*^	101^*^	142
37	8	72	63	105
38	9	41	45	71
39	9	62	70	134
40	9	69	84	97
41	10	56	65	33
42	10	84	85	102
43	11	44	54	71
44	11	72	47	76
45	11	46	48	107
46	11	33	61	75
47	11	69	61	102
48	12	51	44	82
49	12	39	44	54
50	12	55	48	75
51	13	42	72	52
52	13	65	74	97
53	13	41	59	56
54	14	40	26	59
55	15	69	62	93
56	18	44	49	30

*N, number of valid cases; AS, Asperger Syndrome*.

* *values above the upper tolerance limit*.

As a whole group, the ASD participants showed chromatic discrimination deficiency (any type of defect) in 6/20 (30%). In the autistic group, decrements were observed in 3/9 (33%), of whom, 2 revealed a tritan defect, and 1 showed deficits along the 3 confusion lines of the Trivector test (diffuse defect). In the AS group, 3/11 (27%) of the participants were found outside tolerance limits with elevated thresholds along 2 or 3 color confusion axes (Table 3). The ratios and profiles of color vision losses among individuals were not changed by adopting the limits provided by the CCT handbook (100-protan, 100-deutan, and 150-tritan). The Yates' Corrected Chi-Square showed no significant differences in the frequency of color vision losses (*Y* = 0.09, *p* = 0.77) or in the type of defect (tritan or diffuse) (*Y* = 0.75, *p* = 0.38) between the ASD groups.

Within the 6–10 years cluster, AS participants had significant higher thresholds for the protan axis in comparison to participants with autism and controls [KW-H(2.34) = 7.48, *p* = 0.02]. Comparison of color vision thresholds in the 11–19 years old cluster showed no significant differences among the 3 groups (Table 4).

**Table 4:** Comparison of color vision threshold medians among the age groups.

	**Autistic group**	**AS group**	**Control group**	**KW-H**	*p*-value^*^
**6–10 years**					
Protan axis	72 (44–105)	87 (70–129)	64 (33–93)	7.48	0.02
Deutan axis	53 (44–130)	71 (42–457)	62,5 (36–101)	1.80	0.40
Tritan axis	92 (58–323)	107 (46–273)	101 (23–151)	1.10	0.57
**11–19 years**					
Protan axis	43 (35–51)	48,5 (32–63)	45 (33–72)	1.03	0.59
Deutan axis	56 (33–73)	34 (29–50)	51 (26–74)	3.91	0.14
Tritan axis	101 (44–167)	66 (35–93)	75 (30–107)	0.96	0.61

*AS, Asperger Syndrome. Data are medians of the discrimination vector length (range)*.

* p-value obtained by Kruskal-Wallis test

Spearman correlations between color vision thresholds and estimated IQs or Vocabulary/Block Design scaled scores were not statistically significant in any group. However, improvement of color vision performance with age was significant for the three color confusion axes in the case of the AS participants, and for protan and tritan axes within controls (Table 5).

**Table 5:** Spearman correlation coefficients between color vision measurements and IQs, Vocabulary and Block Design scores and ages.

	**Protan**		**Deutan**		**Tritan**	
	***n***	***r_*S*_***	***n***	***r_*S*_***	***n***	***r_*S*_***
**CORRELATION WITH IQ**						
Autism	9	0.35	9	0.15	9	0.14
AS	11	0.35	11	0.05	11	0.26
Control	28	0.10	29	−0.06	29	0.22
**CORRELATION WITH VOCABULARY SCORE**						
Autism	9	0.47	9	−0.09	9	0.25
AS	11	0.38	11	0.11	11	0.08
Control	28	0.05	29	0.07	29	0.08
**CORRELATION WITH BLOCK DESIGN SCORE**						
Autism	9	0.09	9	−0.26	9	0.14
AS	11	0.33	11	0.31	11	0.27
Control	28	0.13	29	−0.01	29	0.26
**CORRELATION WITH AGE**						
Autism	9	−0.40	9	−0.11	9	−0.22
AS	11	−0.89^*^	11	−0.82^*^	11	−0.80^*^
Controls	36	−0.40^*^	29	−0.17	29	−0.55^*^

*N, number of valid cases; IQs, intelligence quotients; AS, Asperger Syndrome*.

* *p < 0.05*.

Five of the 6 ASD participants with higher color vision thresholds showed mean latency in the average range (percentile 30–70%) on the CANTAB RVP as a measure of response speed. The performance of only one participant of the AS group was slightly below the average range (percentile range 20–25%).

## Discussion

Of the overall 20 ASD children and adolescents examined, 6 (30%) had elevated color vision thresholds. The alterations were diffuse defects in 4/20, and tritan defects in 2/20. Color vision losses were distributed in groups with ASDs as follows: elevated color discrimination thresholds were found in 3/9 (33%) participants of the autistic group and in 3/11 (27%) participants of the AS group. While the AS group had only diffuse defects cases, the group with autism had 2/3 cases of tritan defect.

These findings are the result of threshold measurements obtained with a rigorous psychophysical procedure in a computerized color vision test, the Cambridge Color Test, that has been extensively used in clinical populations. They reinforce previous findings, obtained with other methods, that color discrimination may be poorer in ASDs (Ludlow et al., 2006; Franklin et al., 2008, 2010; Heaton et al., 2008).

The present work showed high frequencies of color vision decrements, but no significant differences between the frequencies in the groups with autism and AS. The subgroup of younger children with AS significant higher thresholds for the protan axis in comparison to participants with autism and controls. Since ASD individuals are known to present delayed development of the visual system (reviewed by Simmons et al., 2009), studies with larger samples are necessary to clarify if a possible color vision delay is more frequent within individuals with AS. Regarding the issue of different ASD subgroups (autism without ID vs. AS), there is a possibility that some of the variability in visual processing changes might be accounted for by the existence of different ASD profiles (as reviewed by Muth et al., 2014). In fact, this is the case for other developmental disorders as dyslexia for instance (e.g., Lachmann and Van Leeuwen, 2008). The line of investigation that take into account the AS represent an opportunity to examine the possibility of subgroups within the autism spectrum and to develop interventions that are tuned to the patients‘ needs.

In a previous study, non-verbal IQ was found to be associated to the performance on the Farnsworth-Munsell 100-Hue Test (Cranwell et al., 2015). However, in the present experiment, the color vision defects do not appear to be related to intelligence scores, since correlation analysis showed no statistical association between color vision thresholds and estimated IQs nor Vocabulary and Block Design scaled scores. Superior performance on Block Design is often found among ASD individuals as a consequence of a local/details processing style (reviewed by Muth et al., 2014). Impaired global processing (integration of information into a meaningful whole) accomplished by enhanced local/details processing are known as ASD characteristics, and compose the basis for the weak central coherence theory in autism (Happé, 1999). It should be considered that the detection of the pseudoisochromatic “C” stimulus of the Cambridge Color Test demands global processing and could be lacking or diminished in autism. In the present study, a correlation between Block Design scores and color vision thresholds would allow attributing the color vision losses to difficulty in global processing, if present. The lack of significant correlations between color vision thresholds and its scores among ASD participants makes it unlikely that the poor color vision performance be due to weak central coherence in the detection of the target stimulus. Indeed, ASD participants that performed poorly on the CCT had mean latencies in the RVP within the average range. This result excludes the hypothesis that a generalized slowing might explain the high color vision thresholds of at least 5 of the 6 participants.

Improvement of color discrimination with age was found in AS children who presented a negative correlation between age and color vision thresholds along the 3 confusion axes, and also among control participants in terms of protan and tritan thresholds. Although the same association was not found for participants with autism, this correlation is expected as a function of the development of color vision, in which thresholds follow a U-shaped curve with better performances at late adolescence and young adulthood (Knoblauch et al., 2001; Paramei and Oakley, 2014). In the present study, color vision losses within the AS group were concentrated in children with the lowest age (6 years).

### Color vision losses in ASD

Non-specific deficits of color vision in ASD were found by Franklin et al. (2010) who tested 34 autistic children without ID (11–14 years old) and a control group on the Farnsworth–Munsell 100-hue test and on a chromatic discrimination task involving detection of boundaries between two equiluminant colors. ASD group showed diffuse color vision difficulties on the FM-100 hue test and higher thresholds on the chromatic discrimination task. There were no statistical differences between groups in luminance tasks.

Poorer color discrimination was also described in 13 autistic children and adolescents with ID and also in a group with moderate learning disabilities (both groups with low non-verbal IQs) in comparison to typical developing controls (Heaton et al., 2008). The authors administered 2 tasks integrating color discrimination and color memory. Participants with autism as well as those with moderate learning difficulties differed significantly from controls in a discrimination task in which three colored patches (examples of primary hues of red, blue, green and yellow) were presented on a computer screen, wherein two (distractor patches) differed from a third (target patches) in Munsell hue space. On the other hand, autistic children and adolescents with intellectual disability showed significant better memory for unlabeled color information (patches) paired to familiar pictures (animals) in relation to participants with moderate learning difficulties and typical developing children.

The present findings showed that both the autistic and AS groups display a high proportion of color vision defects—about 30% of the sample studied. Diffuse and tritan defects were found. With this respect, color discrimination deficits on the blue–yellow axis were found to be associated to conditions such as use of medication (Jafarzadehpur et al., 2014), or Attention Deficit Hyperactivity Disorder (ADHD). The reduced chromatic discrimination was attributed to a retinal dopamine hypofunctioning which affected the blue–yellow system (Banaschewski et al., 2006). Despite the findings pointing that ADHD and ASD often co-occur, the previous DSM-4 has not allowed a simultaneous diagnosis of these 2 conditions, which now are recognized by the DSM-5 as comorbid disorders (Leitner, 2014). A differential diagnosis for ADHD is required in order to clarify the nature of color vision deficiency found in the ASD participants with tritan defect in the present study.

Although a previous study has shown a significant positive correlation between performance in the FM100 and nonverbal IQ in young adults with ASD (Cranwell et al., 2015), color vision losses found in the present work cannot be explained by cognitive deficit, since there was no correlation between IQs and color discrimination thresholds. All participants had average or superior IQ scores. In addition, the color vision test used is a very simple psychophysical task and has been successfully employed in other groups of comparable age who also may include lower IQs, such as patients with Duchenne muscular dystrophy (Costa et al., 2007).

### Relating color vision losses and other perceptual alterations in ASD

Although there are only a few studies on color vision in ASD, the literature is filled with investigations on the visual processing of this group. One of the visual symptoms that have been more extensively investigated is the bias toward the processing of local features of stimuli (in detriment of the processing of global features). Typically developing individuals are quicker in identifying global features of stimuli as compared to local ones (as shown for Navon stimuli; Navon, 1977). As some studies have found the opposite for ASD individuals (in tasks such as Embedded Figures, Navon Letters and Coherent Motion, reviewed by Muth et al., 2014) and some authors have proposed a causal relation between this local bias, the changes in face perception and the difficulty to identify emotions in face stimuli (reviewed by Watson, 2013), this symptom was extensively investigated.

To explain the ASD alterations on face perception and responses to emotional stimuli in terms of a local bias in visual processing is controversial for at least two reasons. First, there is recent evidence that a local bias or field independence is not a universal trace of ASD, as it seemed to be present only in western habitants and not in an eastern population (Koh and Milne, 2012). Second, recent meta-analysis studies do not confirm that individuals with ASD generally present different visuo-spatial processing or a local bias (Muth et al., 2014; van der Hallen et al., 2015). Results from different researches are often contradictory and there is no evidence of enhanced local visual processing or global visual processing deficit in ASD. Based on previous findings, authors suggest a general slowing in global processing (van der Hallen et al., 2015). According to Muth et al. (2014), the only tasks where ASD participants were systematically different from controls were Figure Disembedding and Block Design, with a rather small effect size. In fact, Dillen et al. (2015) proposes that there are no differences between ASD adolescents (average 16.7 years old) and controls in an embedded figures task and a configural superiority effect test. Both tasks measure the effect of context clues on target detection and differences in performance might be considered evidence for local or global biases in visual discrimination. As Dillen et al. (2015) found no differences between groups, the authors suggest there is no local bias in ASD adolescents.

In the context of the relevant and recent debate on the nature and extent of visual perception changes in ASD, it is important to discuss how the color vision losses reported here relate to the other visual symptoms in ASD and to the state of the art of this field of research today. Muth et al. (2014) highlights the large heterogeneity between the results found in the meta-analysis. They propose that the large variability between skills and cognitive profiles in the ASD spectrum might account for these differential results. In contrast, the diffuse color vision losses presented here for the AS group is in agreement with investigations of color discrimination in ASD published before. The homogeneity and replicability of this result highlights its relevance for the understanding of visual alterations in ASD.

The pattern of color vision losses found here suggests that, as a sensory measure, these losses are independent of the intelligence level. In many cases, cognitive ability and language skills seem to influence the performance in tasks that integrate color vision discrimination and complex processes such as memory or reading (Ludlow et al., 2006; Heaton et al., 2008). Heaton et al. (2008) suggest the importance of language ability in tasks involving memory and color perceptual discrimination because of the tendency of color naming as a strategy of memorization. Also, identification and memory (delayed memory to sample) of an odd colored patch among distractors were significantly less accurate in children with autism without ID compared to controls (Franklin et al., 2008).

Although in cases as mentioned above, higher order cognitive functions were involved in the tasks, this is not the case for the Cambridge Color Test. This task involves non-significant memory, language or executive functioning demands. Therefore, in face of the lack of correlation between color vision loss and intelligence, we would like to argue that the visual change reported here is very close to a pure and basic visual processing deficit.

There are reports showing no differences between ASD participants and controls in the performance of visual perception tasks, but when the two groups were compared using fMRI, significantly different strategies and brain areas were used by the individuals with ASD (e.g., Lee et al., 2007; Damarla et al., 2010). If differences in brain activation were found in tasks for which there was no behavioral difference, there is a chance that different brain activation might be observed in tasks such as that studied here. Therefore, further studies should address color vision losses in ASD with different and complementary techniques such as imaging, electrophysiology and non-invasive brain stimulation.

Then, in face of the current debates on ASD subgroups and heterogeneity of visual processing deficits, what could the color vision losses presented here mean? Speculatively, the variety of visual processing alterations in ASD could mean that the disorder does not affect one specific area or pathway, but the interaction between different areas and processes. This view has been proposed to explain the diversity of alterations in other complex neurological disorders like Alzheimer's disease and Multiple Sclerosis (for a review see Stam, 2014). The presence of changes in processes and tasks as distinct as color vision, perceptual organization and face perception, supports a more complex and global neurological alteration in ASD.

Finally, the current study has limitations due to its small sample size and to the fact that it cannot ascribe the differences found to specific pathways or mechanisms. Mechanisms for chromatic losses may be either at the retinal level and/or reflect reduced cortical integration, and the present study is not able to provide a definitive basis for the establishment of the origin of the color vision loss. For this reason, the findings must be tested in larger controlled studies in order to verify whether they can be generalized across the broader ASD. Diagnosis or screening of ADHD is desirable in view of the prevalence of comorbidity of ASD and ADHD, and previous findings of color vision defect in the latter. In addition, an evaluation of autistic traits should be performed in the control group, since perceptual alterations observed in ASDs may be found in typically developing individuals with autistic traits (Robertson and Simmons, 2013).

In summary, our results showed that color discrimination was impaired in about 30% of the children/adolescents with ASD, that these losses were diffuse in most cases and that they were similarly prevalent among participants with autism and AS. Sensory alterations are frequent in ASDs and the rationale for more investigation lies on the search for physiopathological mechanisms of the disorder as well as in the possibility of their inclusion in the set of behavioral characteristics that comprise the ASDs.

## Author contributions

EZ: Design of the study and methodology, data collection and analysis, critical review and implementing modifications to the study, writing of the manuscript, and final approval of the version to be published. TC: Data collection and analysis, critical review, writing of the manuscript, and final approval of the version to be published. MB: Design of the methodology, critical review, writing of the manuscript, and final approval of the version to be published. MC: Design of the methodology, data analysis, critical review, writing of the manuscript, and final approval of the version to be published. DB: Critical review, genetic analysis of one individual with ASD, writing of the manuscript, and final approval of the version to be published. DV: Design of the study and methodology, critical review of the study, writing of the manuscript, and final approval of the version to be published.

### Conflict of interest statement

The authors declare that the research was conducted in the absence of any commercial or financial relationships that could be construed as a potential conflict of interest.

## Abbreviations

ASD: Autism spectrum disorder
IQ: Intelligence quotient
HFA: High-functioning autism
DSM: Diagnostic and Statistical Manual of Mental Disorders
AS: Asperger Syndrome
ICD: International Statistical Classification of Diseases and Related Health Problems
WHO: World Health Organization
CARS: Childhood Autism Rating Scale
WISC: Wechsler Intelligence Scale for Children
WAIS: Wechsler Adult Intelligence Scale
ETDRS: Early Treatment Diabetic Retinopathy Study
CCT: Cambridge Color Test
CIE: International Commission on Illumination (Commission Internationale de l'Eclairage).
